# The metabolic network of the last bacterial common ancestor

**DOI:** 10.1038/s42003-021-01918-4

**Published:** 2021-03-26

**Authors:** Joana C. Xavier, Rebecca E. Gerhards, Jessica L. E. Wimmer, Julia Brueckner, Fernando D. K. Tria, William F. Martin

**Affiliations:** Institute for Molecular Evolution, Heinrich-Heine-University, 40225 Düsseldorf, Germany

**Keywords:** Molecular evolution, Bacterial evolution, Phylogeny, Biochemical reaction networks

## Abstract

Bacteria are the most abundant cells on Earth. They are generally regarded as ancient, but due to striking diversity in their metabolic capacities and widespread lateral gene transfer, the physiology of the first bacteria is unknown. From 1089 reference genomes of bacterial anaerobes, we identified 146 protein families that trace to the last bacterial common ancestor, LBCA, and form the conserved predicted core of its metabolic network, which requires only nine genes to encompass all universal metabolites. Our results indicate that LBCA performed gluconeogenesis towards cell wall synthesis, and had numerous RNA modifications and multifunctional enzymes that permitted life with low gene content. In accordance with recent findings for LUCA and LACA, analyses of thousands of individual gene trees indicate that LBCA was rod-shaped and the first lineage to diverge from the ancestral bacterial stem was most similar to modern Clostridia, followed by other autotrophs that harbor the acetyl-CoA pathway.

## Abstract (web summary)

Joana C. Xavier, Rebecca E. Gerhards and colleagues reconstruct the habitat and lifestyle of the last bacterial common ancestor (LBCA) through the construction of the metabolic network and gene tree analysis of 146 LCBA protein families. Their analyses indicate that the LBCA was rod-shaped, and that the first lineage to diverge from the ancestral bacterial stem was most similar to modern Clostridia, followed by other autotrophs that harbor the acetyl-CoA pathway.

## Introduction

Among all cells on Earth^1^, bacteria are not only the most abundant, they comprise the most diverse domain in terms of physiology and metabolism^2^ and are generally regarded as ancient^3–5^. Isotopic signatures trace autotrophy 3.9 billion years back in time^6^. Based on the universality of the genetic code, amino acid chirality, and universal metabolic currencies, there is an agreement that a last universal common ancestor (LUCA) predated the divergence of bacteria and archaea. Because the bacterial and archaeal domains are monophyletic, there is evidence for one clear ancestor for each domain—the last bacterial common ancestor (LBCA) and the last archaeal common ancestor (LACA). Phylogenomic reconstructions indicate that LUCA was a thermophilic anaerobe that lived from gasses in a hydrothermal setting^7^, notwithstanding contrasting views^8,9^. Both phylogenomics and geological evidence indicate that LACA was a methanogen^10–12^, or a similar anaerobic autotroph that fixed carbon via the Wood–Ljungdahl (also known as acetyl-CoA) pathway^12^. Reconstructing the habitat and lifestyle of LBCA is, however, impaired by lateral gene transfer (LGT)^13^, which decouples physiological evolution from ribosomal phylogeny. Like LUCA and LACA, LBCA must have been an anaerobe, because the accrual of atmospheric oxygen occurred much later in Earth’s history, as a product of cyanobacterial metabolism^14–16^. Although some details of Earth’s oxygenation continue to be debated, it is generally accepted that the Great Oxidation Event occurred ~2.4 billion years ago^4,16,17^. The most important difference between anaerobes and aerobes is related to energy; anaerobic pathways such as fermentation, sulfate reduction, acetogenesis, and methanogenesis yield only a fraction of the energy when compared to aerobic pathways^18^, but this is compensated by the circumstance that the synthesis of biomass costs 13 times more energy per cell in the presence of O_2_ than under anoxic conditions. This is because, in the reaction of cellular biomass with O_2_, the thermodynamic equilibrium lies very far on the side of CO_2_. That is, the absence of O_2_ offers energetic benefits of the same magnitude as the presence of oxygen does^19–21^. Although the advent of O_2_ expanded routes for secondary metabolism, allowed novel O_2_-dependent steps in existing biosynthetic pathways, and allowed the evolution of new heterotrophic lifestyles by enabling the oxidation of unfermentable substrates, the advent of O_2_ did not alter the nature of life’s basic building blocks nor did it redesign their biosynthetic pathways^22,23^. It did, however, promote LGT for genes involved in O_2_ utilization^24^. In other words, the fundamentals of biochemistry, metabolism, and physiology were invented in a time when the Earth was anoxic.

Both from the geochemical and the biological standpoint, looking back into the earliest phases of evolution ca. 4 billion years ago is challenging. The geological challenge is that rocks of that age are generally rare, and those that bear traces of life are extremely scarce. The biological challenge is that LGT has reassorted genes across genomes for 4 billion years. As an alternative to reconstructing gene history, metabolic networks themselves harbor independent inroads to the study of early evolution^25^. Metabolic networks represent the set of chemical transformations that occur within a cell, leading to both energy and biomass production^26^. Genome-scale metabolic networks are inferred from a full genome and the corresponding full set of functional (metabolic) annotations^27^, allowing for predictive models of growth and insights into physiology^28^. Furthermore, metabolism itself is connected to the informational processing machine in the cell, because enzymes are coded in DNA, transcribed, and translated, while they also produce the building blocks of DNA and RNA and polymerize them. However, metabolism is much more versatile than information processing. Metabolic networks include multiple redundant paths, and in different species, different routes can lead to the same functional outcome. Because metabolism is far more variable across lineages than the information processing machinery, the genes coding for enzymes are not universal across genomes and are much more prone to undergo LGT than information processing genes are^29^. This circumstance has impaired the use of metabolic enzymes for the study of early prokaryotic evolution.

Metabolic networks and metabolic enzymes unquestionably bear witness to the evolutionary process, but methods to harness their evolutionary information are so far lacking. Here we take a simple but effective approach at inferring the metabolism of LBCA, by focusing on anaerobic genomes and genes that are widely distributed among them. We reconstruct the core metabolic network of LBCA independent of any single backbone phylogenetic tree^30^ for the lineages in question. In doing so, we harness the information in thousands of individual trees for gene families of anaerobic prokaryotes, analyze converging signals, and point to the modern groups most similar, in terms of metabolism, to the groups that diverged earliest from LBCA.

## Results

### Conservation in anaerobic groups unveils LBCA’s physiology

To identify genes tracing to the LBCA, we started from 5443 reference genomes from bacteria and selected those 1089 classified as anaerobic by virtue of lacking oxygen reductases^31^ and having >1000 protein sequences (to exclude energy parasites; Supplementary Data 1 and Supplementary Table 1). The resulting genomes contained 2,465,582 protein sequences that were then clustered into 114,326 families. Of these, 146 families have at least one sequence present in all the 25 major taxonomic groups analyzed. These groups correspond roughly to phyla in GenBank taxonomy, with the exception of Proteobacteria and Firmicutes, which we split into Classes due to their high representation in the dataset. It is worth mentioning that the abundance of Firmicutes and Proteobacteria is not only a result of taxonomic oversampling but is also a reflection of their orders-of-magnitude larger abundance in natural habitats^32^. Upon closer inspection, the families were present in most of the genomes in the analysis, with 122 of the 146 present on average in at least 90% of all genomes in a group (Supplementary Data 2 and Supplementary Fig. 1). These genes are nearly universal and are among the most vertically inherited genes in prokaryotes (Table 1). These 146 families were rechecked manually with regards to functional annotation (Supplementary Data 3) to provide a list of gene functions that trace to LBCA. Around half of those families are involved in information processing, protein synthesis, or other structural functions (Table 1), and the other half can be mapped to at least one metabolic reaction in KEGG, the Kyoto Encyclopedia of Genes and Genomes (even if often also involved in information processing, e.g., the transfer RNA (tRNA) charging category), thus providing insights into LBCA’s physiology and lifestyle.

**Table 1:** Functional categories for the 146 LBCA protein families.

Functional category	Number of protein families	Average family size	Average verticality
Ribosomal proteins	27	1082	12.260
Translation	17	1083	11.803
tRNA charging	16	1058	12.618
DNA recombination and repair	10	1055	13.165
DNA replication	9	1025	12.669
tRNA modification	9	1075	11.036
Transcription	3	1091	16.123
rRNA modification	5	1056	9.513
Carbohydrate and energy metabolism	10	1062	9.422
Protein modification, folding, sorting, and degradation	9	1113	9.727
Lipid and cell wall metabolism	8	1020	9.473
Nucleotide metabolism	7	1073	10.712
Metabolism of cofactors and vitamins	6	901	7.797
Amino acid metabolism	5	917	9.765
Membrane protein targeting	3	984	13.823
Cell division	2	1060	14.946

For each category, the number of protein families annotated, the average family size, and the average verticality (higher meaning less subject to LGT; see “Methods”) are shown.

Various lines of evidence suggest that the first cells were autotrophs that generated acetyl-CoA and pyruvate via the acetyl-CoA pathway^33–35^ and sugars via gluconeogenesis^36–38^. LBCA possessed a nearly complete trunk gluconeogenetic pathway with pyruvate kinase (PK), enolase, phosphoglycerate kinase (PGK), glyceraldehyde 3-phosphate dehydrogenase, and triosephosphate isomerase. Phosphoglycerate mutases, which can be either 2,3-bisphosphoglycerate-dependent or cofactor-independent, escape the criteria of universality, but are highly distributed, the former in 21, the latter in 18 of the 25 bacterial groups sampled. Because the PK reaction is reversible in eukaryotes in vivo^39^ and in bacteria^40^, bacterial PK likely functioned in the gluconeogenetic direction to provide LBCA with phosphoenolpyruvate for amino acid and peptidoglycan synthesis^41^ and carbon backbones with more than three carbon atoms in an early Earth environment rich in CO_2_^42^. Four other kinases in addition to PK and PGK trace to LBCA, two involved in cofactor metabolism and two in phosphorylating ribonucleotides to nucleoside diphosphates, whose further activation to LBCA’s NTPs could have been carried out via substrate promiscuity of PK, as it occurs in anaerobically grown *Escherichia coli*^43^. Also tracing to LBCA are two enzymes involved in cell division, FtsH and FtsY, which however also fulfill a number of other functions in the cell including protein degradation and assembly^44^ and correct targeting of proteins and ribosomes to the membrane^45^. Three other membrane-targeting proteins can be traced to LBCA: Ffh, YidD, and SecA of the sec pathway. One validation of our analysis is the absence of important genes in LBCA’s families that were lost in the ancestor of particular groups, for example, FtsZ, present in only 24 out of 25 of the taxonomic groups in our dataset, consistently with previous reports of its loss in Chlamydiae^46^.

### Only nine compounds were required to complete intermediary metabolism in LBCA

**Fig. 1:**
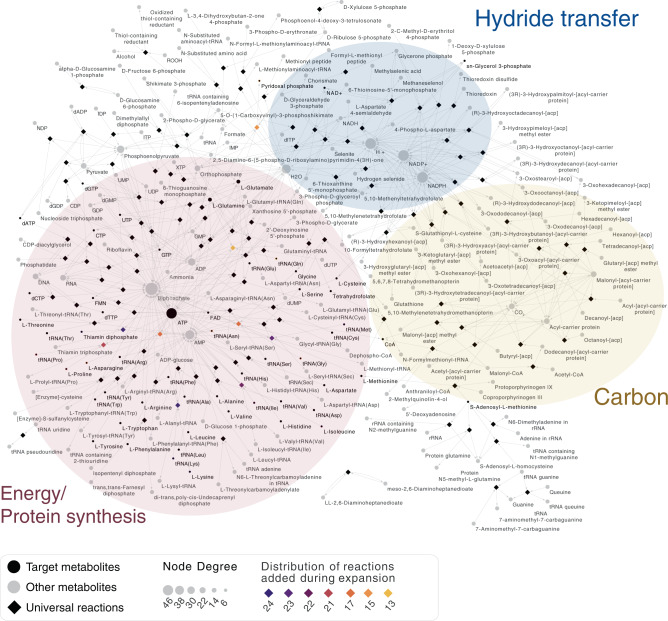
Metabolic network of LBCA expanded with 9 genes to include 57 universal biomolecules. Metabolic interconversions encoded by 146 LBCA genes plus 9 genes present in fewer groups are shown in a bipartite graph, with 243 metabolites (circular nodes) and 130 reactions (diamond nodes). Black circles represent the 57 universal target metabolites and gray circles represent the remaining metabolites. Note, however, that some of these are also universal (e.g., NADH), but directly connected to the chosen targets (e.g., in that case NAD^+^). Node sizes increase according to node degree. Diamonds (reactions) are colored according to the presence of genes encoding for those reactions in different taxonomic groups: in black, reactions present in all taxa; in a gradient from purple to orange reactions added during network expansion and distributed in fewer taxa (target compounds are highlighted with the same outline color if they were introduced with network expansion). Transparent colored ellipses highlight the core of energy (red) hydride transfer (blue) and carbon (yellow) metabolism.

The list of LBCA genes is conservative because our criteria, although not imposing bacterial universality, do require the presence in 25 higher taxonomic groups. However, even though the list is short, the 146 protein families of LBCA generate a tightly connected metabolic network (Supplementary Fig. 2) of 243 compounds with only one reaction (diaminopimelate epimerase) out of 130 disconnected from the rest (Supplementary Data 4A). The network is close to complete in that it generates 48 of the 57 universally essential prokaryotic metabolites^47^: the 20 amino acids, four DNA bases, four RNA bases, eight universal cofactors, glycerol 3-phosphate as a lipid precursor, and 20 charged tRNAs (Supplementary Data 4B). The compounds missing are the charged tRNAs for Lys, Met, Ile, Pro, Asn, Gly, and Gln and two cofactors (thiamine diphosphate and pyridoxal 5-phosphate). Using a network expansion algorithm^48^, adding all reactions encoded by non-LBCA genes to the network, and then sequentially and gradually removing them until the production of all universal metabolites was possible with the minimal set of reactions (see “Methods”), we found that the addition of only nine genes—seven aminoacyl tRNA synthetases (aaRS), ADP: thiamine diphosphate phosphotransferase and d-ribulose 5-phosphate, d-glyceraldehyde 3-phosphate pyridoxal 5′-phosphate-lyase—completes the network to generate all 57 universal compounds (Fig. 1 and Supplementary Data 4). It is likely that ancestors of the two classes of aaRS enzymes acted promiscuously in charging tRNA in LBCA^49^. The network is not self-generated from an initial set of nutrients^50^. It would have required additional genes derived from LUCA^7^ and lost in some lineages of anaerobic bacteria (including transporters, completely absent in the set of 146 genes) and compounds from geochemical synthesis^34,35^ to be a completely functional genome-scale metabolic network. However, the majority of the core of cellular metabolism is represented in the network.

LBCA’s network is highly structured around three major metabolic hubs: (i) ATP/diphosphate, (ii) NADP(H)/H+, and (iii) CO_2_/ACP/malonyl-ACP. These represent the cores of (i) energy, (ii) hydride transfer, and (iii) carbon metabolism of LBCA (Fig. 1). Malonyl-ACP is central in the initiation and regulation of fatty acid biosynthesis^51^. When we remove PK from the set of enzymes, the phosphorylation of dADP to dATP is no longer possible, suggesting that PK may have acted promiscuously in early nucleotide phosphorylation^43,52^. The connectivity of ATP mainly involves tRNA charging and protein synthesis (Fig. 1), which might seem unexpected at first, because ATP is the universal currency in all of the metabolism. In modern anaerobes, although, roughly 90% of the cell’s energy budget is devoted to protein synthesis^21^, and similar appears to have applied to LBCA as well.

### The first lineages to diverge were most similar to modern Clostridia

The deepest split in the bacterial trees can identify lineages and traits that reflect LBCA’s lifestyle. Lineages such as Aquificae and Thermotogae were long considered early branching based on trees of ribosomal proteins and ribosomal RNA (rRNA)^53^, but the ribosome cannot speak to the physiology of LBCA because LGT decouples ribosomal evolution from physiology. LGT is extremely frequent within and between most bacterial groups^13^, it hinders the inference of the bacterial root via traditional phylogenetic analysis by introducing conflicting signals that reduce verticality. To mitigate the effect of LGT, we examined the relative order of emergence for the 25 bacterial groups using 63,324 trees rooted with minimal ancestor deviation (MAD)^54^. In current practice, the majority of root inferences for the domain Bacteria have been done with outgroup rooting^55,56^. Our choice of an outgroup-independent rooting method applied to multiple gene trees is threefold: (i) LGT between Archaea and Bacteria confounds results^13,57,58^; outgroup sequences are notoriously prone to long-branch phylogenetic artifacts^59^; and lack of criteria to assess the quality of different roots, which is possible with MAD. Independent studies have recently shown that the MAD method is more efficient than other rooting methods and robust to a wide spectrum of phylogenetic parameters, both with simulated and empirical prokaryotic gene trees^60^.

**Fig. 2:**
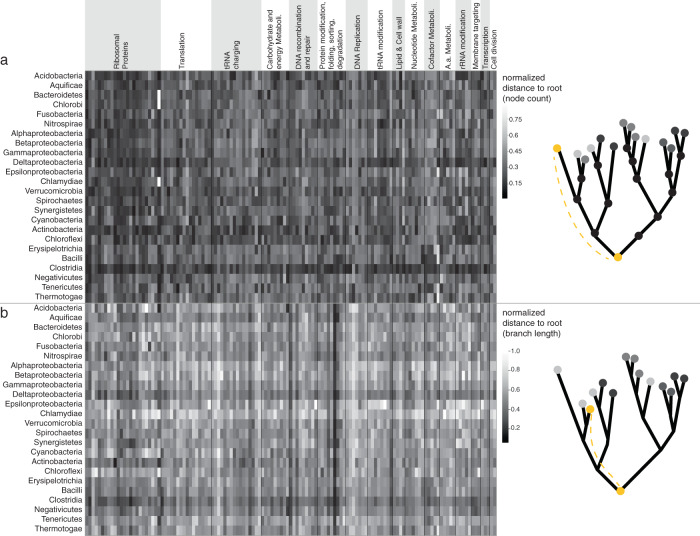
Divergence analyses for 1089 anaerobic genomes using 131 universal trees reveal clostridial species are closer to the root. Analysis of 131 rooted trees of genes universally present in bacterial anaerobic taxa spanning major functional categories (sorted horizontally according to curated classifications shown on top; order as in Supplementary Data 3). Illustrative trees on the side portray the metric used in each analysis and identify the group at the root in each with yellow nodes. **a** Root-to-tip distance measured as node depth (normalized by the largest distance in each tree). **b** Root-to-tip distance measured as branch length (normalized by the largest distance in each tree).

We started by focusing on the trees for the 146 LBCA protein families, and we analyzed the divergence accumulated from the bacterial root to each modern genome, measured as root-to-tip distance in terms of (i) sequence divergence (branch length) and (2) node depth (Fig. 2) (15 trees with ambiguous root inferences were discarded; root ambiguity indexes given in Supplementary Data 3; see “Methods”). The results identify clostridial genomes as the least diverged both in terms of sequence divergence (Wilcoxon’s signed-rank test with Bonferroni correction, largest *p* value < 1e − 5, average normalized distance 0.299) and node depth (Wilcoxon’s signed-rank test with Bonferroni correction, largest *p* value < 0.05, average normalized distance 0.116; Supplementary Fig. 3), followed by Deltaproteobacteria (average normalized divergence 0.354, and average normalized depth 0.156). Anaerobic members of Aquificae also show significant proximity to the root as judged by branch length (average normalized distance 0.382, Supplementary Fig. 3). There are only three genomes of (anaerobic) Aquificae in our dataset, and all three belong to chemolithoautotrophs isolated from hydrothermal vents that can grow on H_2_ and CO_2_^61^. The divergence values for all genomes in all trees ranked from least to most distant show that the top-ranking 12 genomes are all thermophilic species belonging to the class Clostridia, several possessing the acetyl-CoA pathway (Supplementary Table 2). The results shown in Fig. 2 are not dependent on genome abundance in the dataset (the most abundant group is Bacilli, with 38% of all genomes; Supplementary Table 1).

**Fig. 3:**
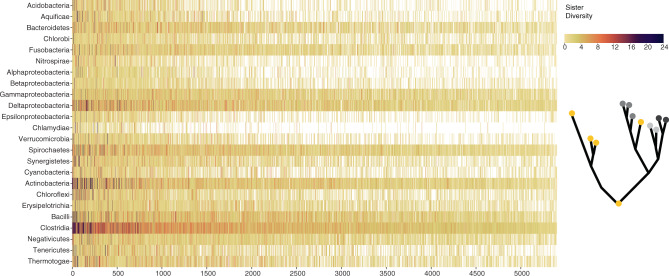
Sister diversity analysis of 5402 phylogenetic trees reveals Clostridia is the most ancestral group. Sister diversity (maximum number of different groups in the sister clade) for each group (rows) for 5402 trees with at least six groups (columns). An illustrative tree portrays the question asked in the analyses, where the yellow group is the one with the highest sister diversity score and therefore inferred as most ancestral.

Prokaryotic gene trees differ from the species tree due both to random phylogenetic errors and to the cumulative impact of LGT^62^. In the absence of LGT, gene lineages branch together (monophyletic) and the phylogenetic diversity of sister clades reflects the time since their origin, with older lineages having higher sister diversity. In the context of gene evolution with LGT, gene lineages branch into multiple clades, with the number of clades increasing with gene transfer prevalence. Because LGT is a continuous phenomenon in prokaryotic evolution, the taxonomic labels of sister lineages change dynamically, but their phylogenetic diversity gives us the means to infer the relative timing for the origin of lineages. To integrate the information of sister relation from all gene trees spanning the 25 bacterial groups, we scored the phylogenetic diversity for sister clades of each group in the individual trees permitting as many inter-group LGT as necessary in the trees (5402 trees with at least six groups, Fig. 3 and Supplementary Data 5). The analyses show Clostridia as the group with the highest sister clade diversity, measured as the maximum number of phyla in a sister clade (on average five), followed by a tie between Deltaproteobacteria, Bacilli, Actinobacteria, and Spirochaetes all with three distinct groups on average present in sister clades. The result stands when looking at the 131 universal trees only, where Clostridia has on average nine distinct sister groups, followed by Actinobacteria with seven and Deltaproteobacteria with five (Supplementary Data 6). Maximum-likelihood ancestral state reconstructions using 131 universal trees indicate that LBCA was a rod-shaped cell (Supplementary Fig. 4) and reconstructs Clostridia as the most ancestral lineage (Supplementary Fig. 5) in agreement with the previous analyses.

**Fig. 4:**
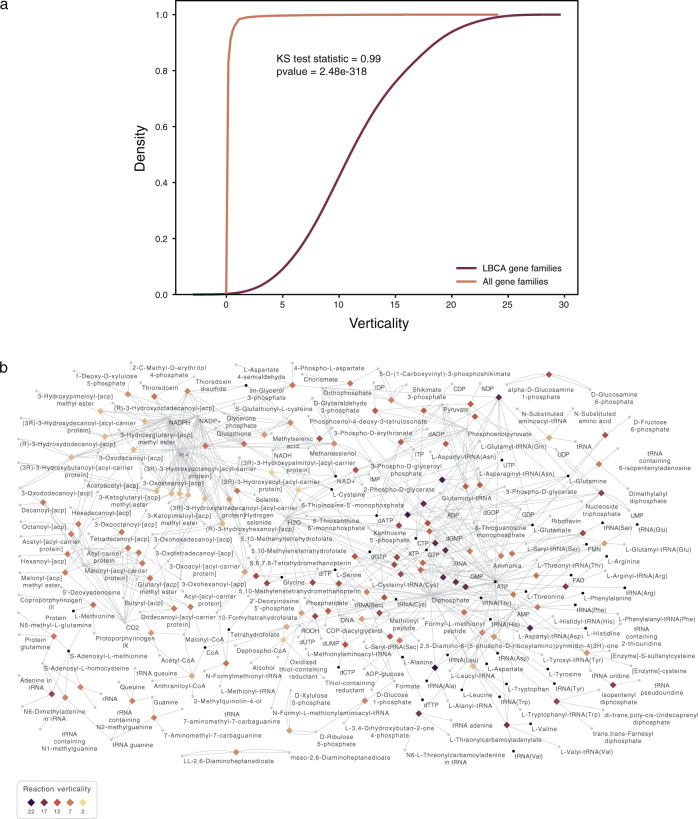
Analysis of verticality for LBCA gene families. **a** Verticality for all prokaryotic gene families (light brown) and for LBCA gene families (dark brown) and Kolmogorov–Smirnov statistics between the two distributions. **b** LBCA metabolic network annotated with verticality value for each reaction node.

The analyses so far suggest that the 146 protein families conserved in all groups of anaerobic bacteria were present in LBCA, not only due to their ubiquitous and nearly universal nature (Supplementary Fig. 1) but also because they form a functional unit: a highly connected, nearly complete core metabolic network (Fig. 1). But is the ubiquitous nature of these genes caused by their antiquity, or is it the result of LGT? To address this question, we obtained all values of verticality for prokaryotic gene families^29^ as a proxy to measure the gene’s tendency to undergo or resist LGT. LBCA’s protein families are distinctively and significantly (Kolgomorov–Smirnov statistic = 0.99, *p* value = 2.4e – 318) more vertical than the average prokaryotic protein family (Fig. 4a, Supplementary Data 7, and Table 1). The metabolic network annotated with verticality values shows that genes involved both in metabolism and information processing (as aaRSs) are highly vertical (Fig. 4b and Supplementary Data 7). Although the most vertically evolving genes in prokaryotic genomes, those for ribosomal proteins, are not involved in specific biosynthesis and hence not represented in metabolic maps, the metabolic functions most closely associated with protein synthesis, those of aaRSs, build the core of a metabolic network that is vertical in nature and thus ubiquitous due to antiquity, not transfer (Fig. 4) and hence ancestral to the domain Bacteria.

## Discussion

By investigating the genomes of anaerobic bacteria, we were able to obtain inferences about the metabolism and physiology of LBCA. Our results indicate that LBCA was autotrophic, gluconeogenetic, and rod-shaped. Our analyses of trees for all genes, not just those universally present in all genomes, point to Clostridia (a class within the phylum Firmicutes) as the modern bacterial group most similar to the first lineages, which diverged from LBCA. This result contrasts with previous analyses placing other groups at the root based on concatenated protein phylogeny^53,56,63,64^, but it is consistent with early proposals based on the evolution of tetrapyrrole synthesis^65^, with studies that place the broader taxon of Firmicutes deep-branching in bacterial trees^37,66^ and with the proposal of a rod-shaped Gram-positive ancestor for bacteria^67^, and, more recently, for Firmicutes^68^. Why do our inferences on the root of the bacterial tree contrast with different roots^63,64^ proposed in other recent analyses? First, our results are based on genome data for cultured organisms with high-quality and complete genomes, and are therefore independent of binning procedures inherent to metagenomic data^69^. In addition, our data are based on genomes for anaerobic bacteria available to date, and is thus less prone to LGT effects associated with the rise of oxygen^24^. The assumption that LBCA was anaerobic is supported by geochemical^14,17^ and phylogenomic^4,16,24^ evidence, and it undoubtedly reduces phylogenetic noise that would be introduced with late-coming aerobic sequences. Furthermore, our results do not rest upon one or two branches in a single concatenated or consensus tree based on ribosomal sequences, an approach that notwithstanding long tradition has strong potential problems^30^, not the least of which is that with concatenated alignments, different methods give fully resolved but conflicting trees, making the results dependent on ad hoc site filtering procedures and specific maximum-likelihood parameters^70^.

Our results are internally consistent, based on the convergence of signals from multiple individual trees for individual protein families (with statistical support, Supplementary Fig. 3). In addition, the core set of 146 families trace to LBCA through multiple lines of evidence: (i) the families are universally present in all taxonomic groups analyzed, and (ii) nearly universally present in all genomes analyzed (Supplementary Fig. 1); (iii) they enable a highly connected and nearly complete core metabolic network (Fig. 1); (iv) they are enriched in information processing genes, known to be ancient (Table 1); (v) their functional repertoire (including RNA modifications, multifunctionality, and gluconeogenesis-early) is in accordance with independent studies for LUCA^7^ and LACA^12,37^; and (vi) they are among the most vertical genes known (Table 1, Supplementary Data 7, and Fig. 4). The metabolic network enabled by the 146 LBCA genes can be completed for universal essential metabolites with only nine genes, all nine of which are present both in Clostridia and Deltaproteobacteria (Supplementary Data 2).

It has been proposed that Gram-negative bacteria originated from Gram-positive bacteria by an early sporulation event^71^, a hypothesis that is compatible with our results. Endospore formation is specific to Firmicutes, implying that if sporulation was an ancient trait, it was subsequently lost before the divergence of most other anaerobic lineages. Spores could have survived in the geologically challenging environments of early Earth^3^, and the loss of sporulation in more moderate environments is facile^72^.

Other groups showing proximity to the root in the phylogenomic tests we performed are Deltaproteobacteria (all tests), three anaerobic species of Aquificae that are significantly closer to the root by branch length (Figs. 2 and 3 and Supplementary Fig. 3) than other lineages, and Actinobacteria, which rank higher than both Deltaproteobacteria and Aquificae in the sister diversity analysis (Fig. 3). What do these groups have in common? Members of all have the acetyl-CoA pathway for carbon fixation and/or energy metabolism^73^; the only carbon fixation pathway present in both archaea and bacteria that traces to LUCA^7^ and that is also present in methanogens, the root of the archaeal tree^10–12^. This physiological trait links LBCA both to LUCA and LACA, and also to anaerobic H_2_-dependent growth in hydrothermal environments^7^. Whereas most Deltaproteobacteria use the acetyl-CoA pathway solely for carbon fixation while reducing sulfate for energy metabolism, recent reports show that some members can use the acetyl-CoA pathway for ATP supply as well^74,75^. The divergence patterns herein inferred are fully consistent with the observation that both Clostridia and Deltaproteobacteria are known to be remarkably polyphyletic. Recently, a proposal to divide Deltaproteobacteria into new phyla has been published, confirming that sulfate/sulfite reduction within the class is ancient^76^. Deep-branching Actinobacteria with the Wood–Ljungdahl pathway have recently been uncovered in serpentinizing systems^77^. In terms of physiology, the acetyl-CoA pathway is undoubtedly an ancient biochemical route^78^. By the measure of analyses presented here, several lineages that use it for survival appear to be ancient as well. The reconstruction of LBCA’s metabolism reveals the presence of several multifunctional enzymes, reducing the number of genes required for its viability, an important evolutionary consequence of ancestral enzyme promiscuity^79^ and possibly a general strategy among the earliest prokaryotes. The physiology of LBCA reconstructed from anaerobes reveals traits well suited to the inhospitable environment of the early Earth^42^.

## Methods

### Data collection and clustering

Bacterial genomes were collected from NCBI, version September 2016^80^. Genomes were classified as anaerobic or aerobic as done elsewhere^31^, rendering 1089 bacterial genomes from anaerobes. Briefly, a dataset of 1784 sequences labeled as heme-copper oxygen reductases (HCOs) and nitric oxide reductases (NORs) was blasted against our dataset of prokaryotic genomes. If one homolog (>25% identity, *e* value <10^−10^, coverage of at least 300 amino acids) for HCOs and NORs was found, the genome was classified as aerobic.

Genomes were assigned their corresponding phyla in NCBI taxonomy, except for (i) Firmicutes and Proteobacteria (the size of which exceeded other phyla by an order of magnitude) where species were assigned to classes for resolution, and (ii) phyla with fewer than 5 species, assigned to “Other Bacteria.” Pairwise local alignments for all protein sequences were calculated with a reciprocal blastp (BLAST+ version 2.5.0)^81^, followed by the calculation of global identities with an adaptation of EMBOSS needle^82^. Pairs of sequences with a minimum global identity of 25% and an *e* value ≤1E − 10 were then used to create protein families with the MCL algorithm^83,84^. For the creation of protein families with the MCL algorithm, the parameters --abc -P 180000 -S 19800 -R 25200 were used, resulting in 114,326 families. Of these, 64,149 were present in at least three species and at least four genomes, and were retained for further analyses.

### Functional annotation

All protein sequences were aligned against the KEGG Orthology (KO) database^26^ (accessed August 2017) using BLAST searches. The best query-subject hits as judged by *E* value, query coverage, and length ratio (cut-off: query coverage ≥80%, *E* value ≤1E − 10, and length ratio between 0.7 and 1.3) were used to annotate the protein sequences individually. We assigned the functional category to each gene family according to the most frequent annotation for the protein sequences in the family. If two or more functional categories occurred with the same frequency, the gene family was annotated within all equally supported categories. For the 146 universal protein families, the annotation of each family in its corresponding functional categories was rechecked manually (Supplementary Data 3).

### Sequence alignment, tree reconstruction, and root inferences

For each gene family, the protein sequences were aligned using MAFFT (Multiple Alignment with Faster Fourier Transform) version 7.130^85^ (parameters: --maxiterate 1000 --localpair; alignments not predictable this way were constructed using the parameter --retree 2). The resulting alignments were used to reconstruct maximum-likelihood trees with RAxML version 8.2.8^86^ (parameters: -m PROTCATWAG -p 12345). Trees were rooted with MAD^54^. Trees with more than one possible MAD root were ignored, leaving 63,324 trees for the subsequent analyses (available in Supplementary Data 5).

### Tree analysis

#### Divergence analysis

To quantify divergence since the LBCA split for each bacterial genome, we calculated root-to-tip distances for all tips in all gene trees measured as (i) the sum of branch lengths (phenetic distance) along the path connecting each operational taxonomic unit to the root and (ii) the sum of branch splits (node depth). To allow for comparisons among trees we normalized the root-to-tip distances for each tree according to the largest distance attained in the tree, so that distance values are bound to the unity interval, with large values indicating more divergence. We scored divergence values to each taxonomic group across all the trees according to the affiliated genome with the smallest root-to-tip distance, independently for each metric (phenetic and node depth). All analyses were performed with custom Python scripts using the Environment for Tree Exploration^87^ (ETE3, version 3.1.1).

#### Sister diversity

We analyzed the distribution of sister relationships for each taxonomic group across the rooted trees as follows: for a given tree with the leaves labeled according to the taxonomic group, we retrieved the set of pure clades for each taxonomic group represented by at least one species in the tree. Note that even though some taxa may not branch as a single clade in the tree, the minimal set of pure (monophyletic) clades can be identified. For each pure clade, the number of taxonomic groups present in the sister clade was recorded (a value in the range of [1–24]) and the sister clade with maximal diversity (in terms of the number of taxonomic groups) was used as sister diversity score. All analyses were performed with custom Python scripts using ETE3^87^ (version 3.1.1).

#### Verticality

All 261,058 values of verticality for all prokaryotic gene families were obtained from Nagies et al.^29^, where the highest possible value is 24 and the lowest is zero. All LBCA protein families were ranked from most to least vertical (Supplementary Data 7). For reactions encoded by multiple protein families, the average value of verticality was taken.

### LBCA metabolic network

#### Network construction

For all 6164 anaerobic bacteria KOs the respective reactions were downloaded from the KEGG reaction database^26^ (version 16-08-2019), 2414 KOs had at least one reaction associated, resulting in 3550 reactions. Reaction reversibility was determined by parsing KGML (KEGG Markup Language) files from 165 KEGG pathway maps. Reactions that did not occur in the KGML files were assigned as irreversible. Seventy-three reactions containing ambiguous stoichiometries (characters n and m) or unknown compounds were discarded. The final set consisted of 3477 reactions.

#### Metabolic network expansion

Twenty proteinogenic amino acids, four DNA bases, four RNA bases, eight universal cofactors, one lipid, and 20 uncharged tRNAs were investigated as targets in the network. The algorithm^48^ started with a complete reaction network containing all 3477 LBCA candidate reactions regardless of their taxonomic distribution. A score was assigned to each reaction, reflecting the likelihood of their presence in LBCAs metabolic network. Reactions with low distribution among taxonomic groups were scored lower, whereas the score increased with the higher taxonomic distribution. The reactions were sorted increasingly by their score. Starting with low scores, reactions were removed temporarily from the full network sequentially. If neither the presence of the target compounds nor the core network was violated, the respective reaction was removed permanently. The reduction algorithm stopped when no further reaction could be removed. The network was visualized with Cytoscape^88^ (version 3.7.2).

#### Ancestral state reconstruction

Ancestral state reconstruction for cell shape and taxonomic groups was performed with PastML^89^ version 1.9.20 using the 131 trees with all taxonomic groups as independent estimates of the prokaryotic phylogeny. The underlying metadata for the tip states was downloaded from JGI GOLD^90^ v.6. The maximum-likelihood-based prediction method MPPA (marginal posterior probabilities approximation) with model F81 was used to reconstruct the states at the root of each tree. The reconstructed states at the root of the trees occurring in the highest frequencies were considered the most likely state for LBCA.

#### Statistics and reproducibility

Statistical tests were performed to assess differences of root-to-tip distances between all 276 possible taxon pairs. For a given taxon pair *a* and *b*, all 131 trees with all taxonomic groups were used and the representative species with smallest root-to-tip distance were recorded for each tree resulting in two distance vectors *D*_*a*_ and *D*_*b*_. Statistical tests were performed with one-sided Wilcoxon’s signed-rank test for paired samples, such that:

H_0_: *D*_*a*_ = *D*_*b*_

H_1_: *D*_*a*_ < *D*_*b*_

Across all taxon pairs, the tests generated a *p* value matrix (24-by-24), and *p* values were considered significant <0.05 after Bonferroni correction (Supplementary Fig. 3). The tests were conducted using the scipy.stats^91^ implementation of the Wilcoxon’s signed-rank test in Python. The Kolmogorov–Smirnov test used to measure significance in the comparison of verticalities was also conducted with the default parameters in the scipy.stats implementation in Python. No random sampling was made in the analyses conducted in this paper.

### Reporting summary

Further information on research design is available in the Nature Research Reporting Summary linked to this article.

## Supplementary information

Supplementary Information

Description of Additional Supplementary Files

Supplementary Data 1

Supplementary Data 2

Supplementary Data 3

Supplementary Data 4

Supplementary Data 5

Supplementary Data 6

Supplementary Data 7

Reporting Summary

## Data Availability

Sequence data that supports the findings of this study are available in NCBI RefSeq^80^ (GCF identifiers used are provided in Supplementary Data 1). Metabolic data is available in KEGG^26^. Metadata is available from JGI GOLD^90^. Phylogenetic trees and all other relevant data are provided as Supplementary Datasets.

## Code availability

All data sources, software packages, and their usage are described in the “Methods” with the corresponding versions and references, including NCBI, KEGG, JGI GOLD v. 6, BLAST v. 2.5.0, EMBOSS needle, MAFFT v. 7.130, RAxML v. 8.2.8, MCL, MAD, ETE3 v. 3.1.1, PastML v. 1.9.20, and Cytoscape v. 3.7.2. New codes used here consisted of batch subroutines to run the aforementioned algorithms multiple times, calculations, and statistical analyses thoroughly described in the “Methods”. The data and results presented in this paper do not result from new software development.
