# Supplementary material for: The metabolic network of the last bacterial common ancestor
Source: Commun Biol. 2021 Mar 26;4:413. doi: 10.1038/s42003-021-01918-4 (PMC7997952; doi:10.1038/s42003-021-01918-4)
Supplement: Supplementary file 1 — Supplementary Information [file 42003_2021_1918_MOESM1_ESM.pdf]

# Supplementary Information

## The Metabolic Network of the Last Bacterial Common Ancestor

Joana C. Xavier[1*#], Rebecca E. Gerhards[1#], Jessica L. E. Wimmer[1], Julia Brueckner[1], Fernando D. K. Tria[1], William F. Martin[1]

[1] Institute for Molecular Evolution, Heinrich-Heine-University, 40225 Düsseldorf, Germany

*e-mail: xavier@hhu.de; [#]these authors contributed equally

**Supplementary Table 1│Bacterial genomes per taxonomic group.** For each taxonomic group, the number of genomes present in the dataset with no filters, after filtering out small genomes (<1000 sequences) and after filtering out the aerobic genomes are shown.

| Taxonomic group | Unfiltered | No small genomes | No small genomes and no aerobes |
|---|---|---|---|
| Gammaproteobacteria | 1,565 | 1,513 | 123 |
| Bacilli | 1,012 | 1,012 | 416 |
| Actinobacteria | 593 | 591 | 110 |
| Betaproteobacteria | 493 | 484 | 47 |
| Alphaproteobacteria | 460 | 415 | 9 |
| Epsilonproteobacteria | 253 | 253 | 3 |
| Bacteroidetes | 185 | 174 | 47 |
| Clostridia | 169 | 169 | 165 |
| Tenericutes | 141 | 18 | 18 |
| Chlamydiae | 113 | 14 | 10 |
| Cyanobacteria | 94 | 94 | 2 |
| Deltaproteobacteria | 74 | 73 | 23 |
| Spirochaetes | 66 | 50 | 34 |
| Thermotogae | 32 | 32 | 32 |
| Other bacteria | 29 | 28 | 18 |
| Deinococcus-Thermus | 26 | 26 | 0 |
| Chloroflexi | 26 | 26 | 17 |
| Fusobacteria | 20 | 20 | 20 |
| Aquificae | 16 | 16 | 3 |
| Chlorobi | 12 | 12 | 7 |
| Negativicutes | 12 | 12 | 12 |
| Verrucomicrobia | 10 | 9 | 3 |
| Acidobacteria | 8 | 8 | 1 |
| Planctomycetes | 8 | 8 | 0 |
| Erysipelotrichia | 8 | 8 | 8 |
| Nitrospirae | 8 | 8 | 1 |
| Acidithiobacillia | 5 | 5 | 0 |
| Synergistetes | 5 | 5 | 5 |
| **Total** | **5443** | **5083** | **1089** |

**Supplementary Table 2 | List of 40 genomes closest to the bacterial root by sequence divergence (branch length) for all trees.**

| Genome ID | Average smallest distance (branch length) | Taxonomic group | Species name |
|---|---|---|---|
| GCF_000175295.2 | 0,4853 | Clostridia | 'Thermoanaerobacter brockii subsp. finnii Ako-1' |
| GCF_000019085.1 | 0,4856 | Clostridia | 'Thermoanaerobacter pseudethanolicus ATCC 33223' |
| GCF_000148425.1 | 0,4918 | Clostridia | 'Thermoanaerobacter sp. X513' |
| GCF_000019065.1 | 0,4918 | Clostridia | 'Thermoanaerobacter sp. X514' |
| GCF_000092965.1 | 0,4919 | Clostridia | 'Thermoanaerobacter mathranii subsp. mathranii str. A3' |
| GCF_000147695.2 | 0,4950 | Clostridia | 'Thermoanaerobacter wiegelii Rt8.B1' |
| GCF_000763575.1 | 0,4957 | Clostridia | 'Thermoanaerobacter kivui strain=LKT-1' |
| GCF_000025645.1 | 0,4972 | Clostridia | 'Thermoanaerobacter italicus Ab9' |
| GCF_000144645.1 | 0,5039 | Clostridia | 'Thermosediminibacter oceani DSM 16646' |
| GCF_000092945.1 | 0,5062 | Clostridia | 'Thermincola potens JR' |
| GCF_000145615.1 | 0,5096 | Clostridia | 'Thermoanaerobacterium thermosaccharolyticum DSM 571' |
| GCF_000144695.1 | 0,5099 | Clostridia | 'Acetohalobium arabaticum DSM 5501' |
| GCF_000189775.2 | 0,5150 | Clostridia | 'Thermoanaerobacterium xylanolyticum LX-11' |
| GCF_000328545.1 | 0,5154 | Clostridia | 'Thermoanaerobacterium thermosaccharolyticum M0795' |
| GCF_000007085.1 | 0,5214 | Clostridia | 'Caldanaerobacter subterraneus subsp. tengcongensis MB4' |
| GCF_000328625.1 | 0,5265 | Clostridia | 'Halobacteroides halobius DSM 5150' |
| GCF_000020485.1 | 0,5280 | Clostridia | 'Halothermothrix orenii H 168' |
| GCF_000184925.1 | 0,5280 | Clostridia | 'Ruminiclostridium thermocellum DSM 1313' |
| GCF_001692755.1 | 0,5287 | Clostridia | 'Ruminiclostridium thermocellum DSM 2360' |
| GCF_000213235.1 | 0,5290 | Clostridia | 'Tepidanaerobacter acetatoxydans Re1' |
| GCF_000255615.2 | 0,5291 | Clostridia | 'Ruminiclostridium thermocellum AD2' |
| GCF_000328765.2 | 0,5292 | Clostridia | 'Tepidanaerobacter acetatoxydans Re1 [redundant no. 2]' |
| GCF_000015865.1 | 0,5300 | Clostridia | 'Ruminiclostridium thermocellum ATCC 27405' |
| GCF_000299355.1 | 0,5303 | Clostridia | '[Clostridium] acidurici 9a' |
| GCF_000214435.1 | 0,5306 | Clostridia | 'Desulfotomaculum nigrificans CO-1-SRB' |
| GCF_000014125.1 | 0,5309 | Clostridia | 'Clostridium novyi NT' |
| GCF_000092345.1 | 0,5325 | Clostridia | 'Clostridium botulinum F str. 230613' |
| GCF_000017045.1 | 0,5332 | Clostridia | 'Clostridium botulinum A str. Hall' |
| GCF_000017025.1 | 0,5342 | Clostridia | 'Clostridium botulinum A str. ATCC 19397' |
| GCF_001483965.1 | 0,5348 | Bacilli | 'Carnobacterium sp. CP1' |
| GCF_000063585.1 | 0,5354 | Clostridia | 'Clostridium botulinum A str. ATCC 3502' |
| GCF_000253195.1 | 0,5358 | Clostridia | 'Clostridium botulinum H04402 065' |
| GCF_000017065.1 | 0,5363 | Clostridia | 'Clostridium botulinum F str. Langeland' |
| GCF_000172575.2 | 0,5365 | Bacilli | 'Enterococcus faecalis OG1RF' |
| GCF_000012865.1 | 0,5366 | Clostridia | 'Carboxydothermus hydrogenoformans Z-2901' |
| GCF_000317975.2 | 0,5366 | Bacilli | 'Carnobacterium maltaromaticum LMA28' |
| GCF_000829015.1 | 0,5371 | Clostridia | 'Clostridium botulinum strain=111' |
| GCF_000007625.1 | 0,5373 | Clostridia | 'Clostridium tetani E88' |
| GCF_000018325.1 | 0,5376 | Clostridia | 'Alkaliphilus oremlandii OhILAs' |
| GCF_000022765.1 | 0,5381 | Clostridia | 'Clostridium botulinum A2 str. Kyoto' |

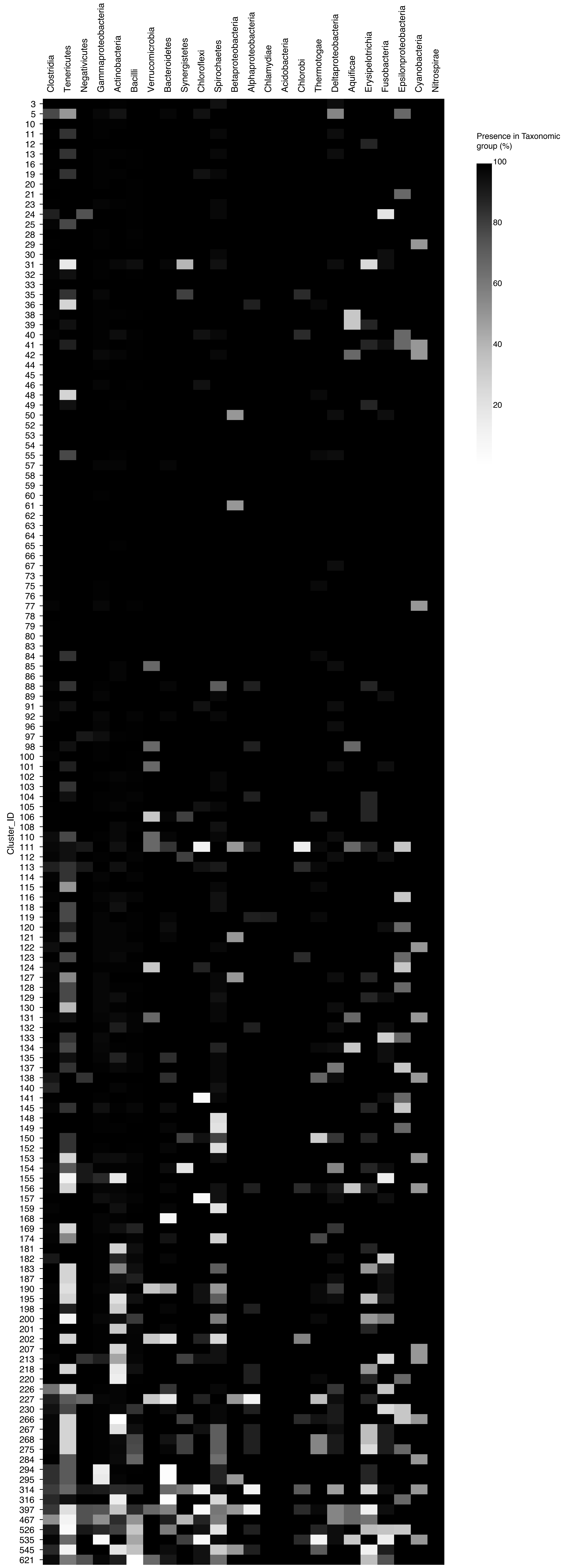


**Supplementary Figure 1 | Taxonomic distribution of LBCA protein families.** 146 protein families (rows) in all taxonomic groups (columns). The color in each cell indicates the proportion of the group (%) where the protein is present according to the scale bar.

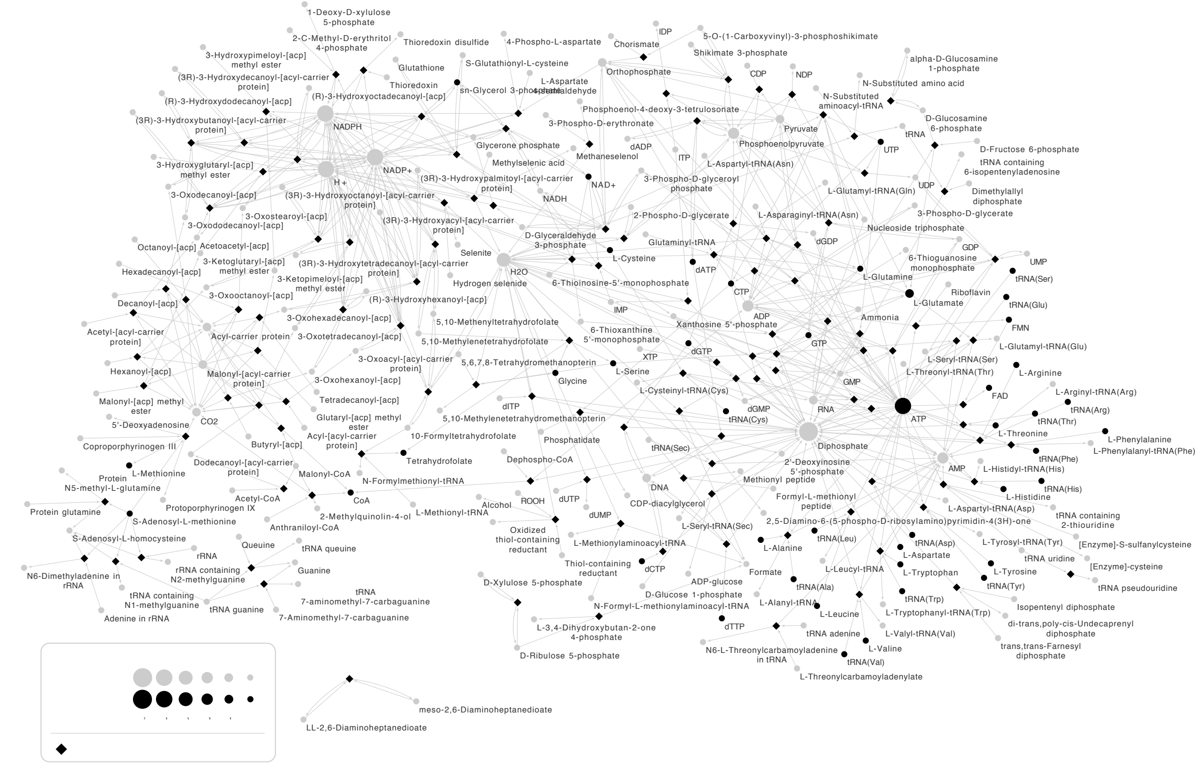


**Supplementary Figure 2 – LBCA's metabolic network before expansion.** Metabolic interconversions encoded by 146 LBCA genes are shown in a bipartite graph, with 224 metabolites (circular nodes) and 121 reactions (diamond nodes). Black circles are the 57 universal target metabolites and grey circles are the remaining metabolites (note that some of these are also universal (e.g. NADH) directly connected to the chosen targets (e.g. in that case NAD+)). Node sizes grow according to degree.

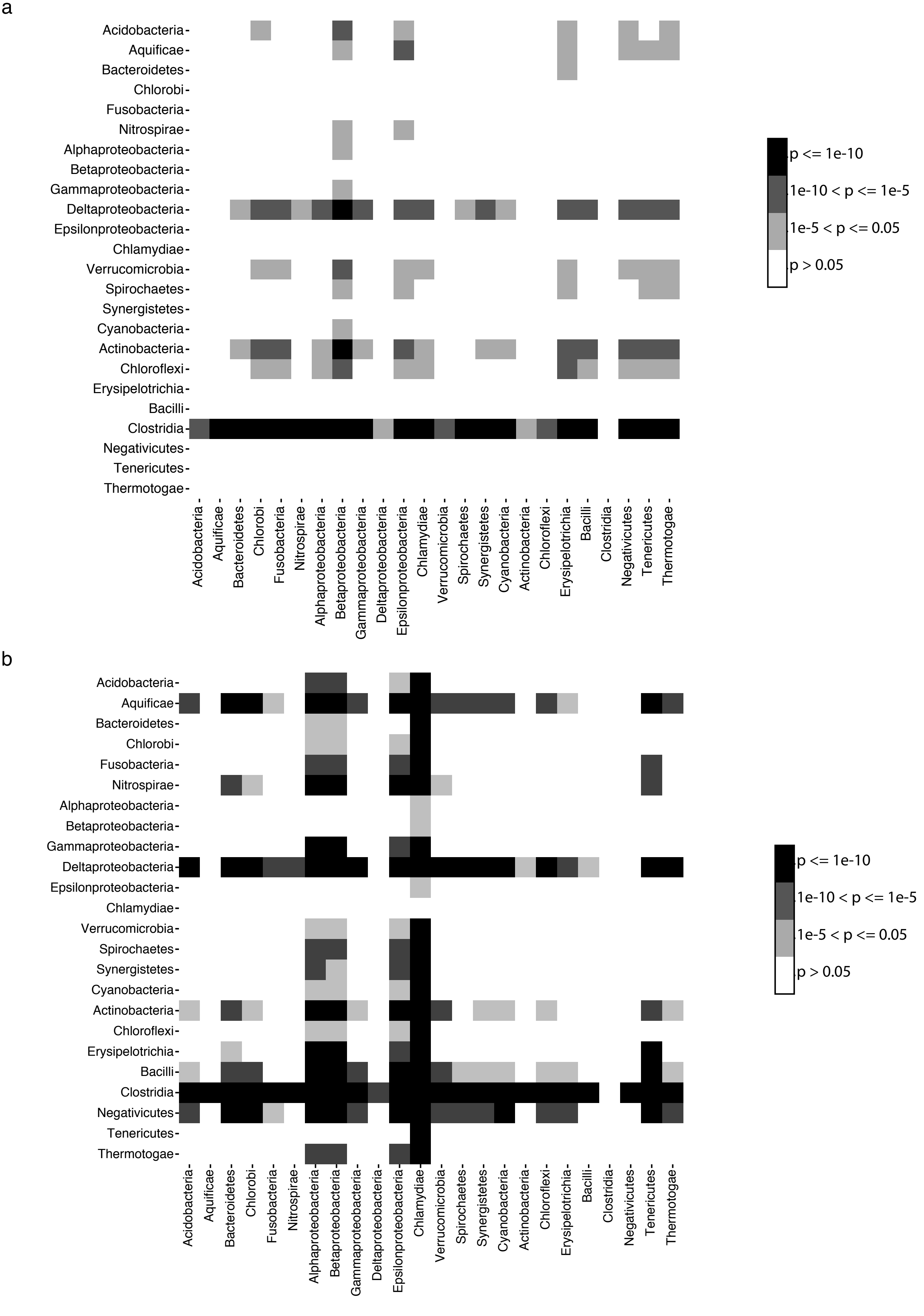


**Supplementary Figure 3 – Statistical tests on divergence analysis for all groups and 131 universal trees.** Metrics were a) node depth and b) branch length (see Methods section).

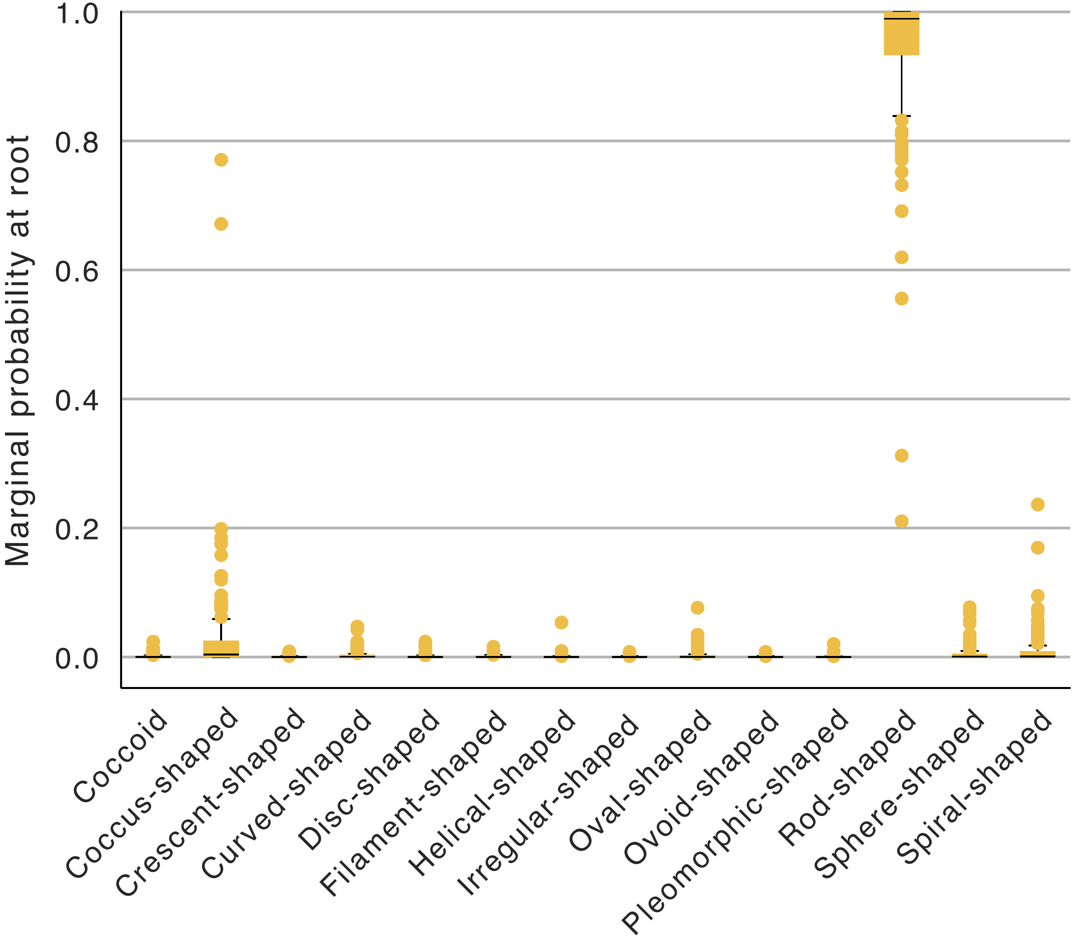


**Supplementary Figure 4 – Marginal probabilities for each cell-shape at the root of 131 universal trees.** Reconstructions by Maximum Likelihood (see Methods).

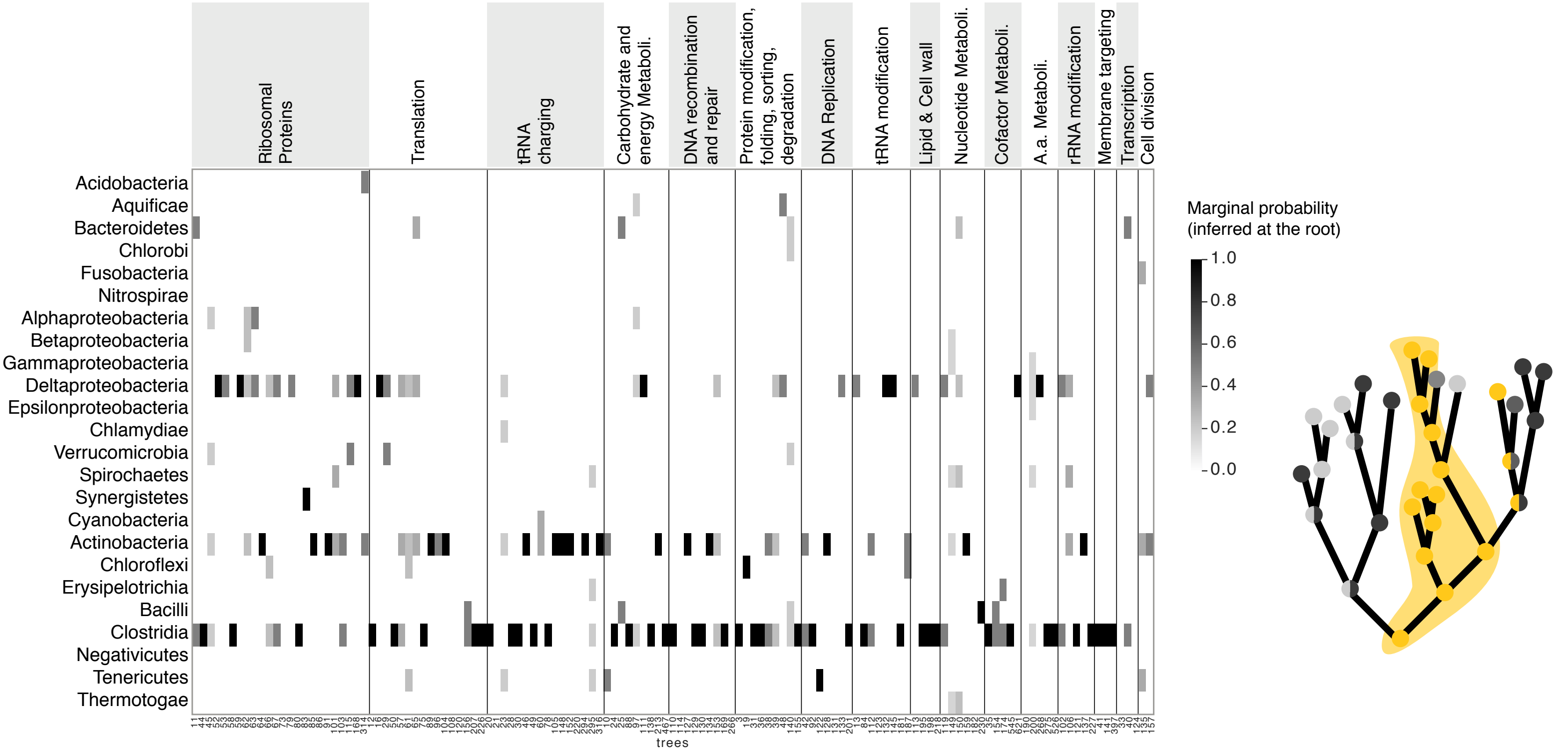


**Supplementary Figure 5 – Marginal probabilities for each taxonomic group at the root of 131 universal trees.** Presence-absence pattern shows marginal probabilities for each group (rows) and each tree (columns). Reconstructions by Maximum Likelihood (see Methods). An illustrative tree portrays the question asked in the analysis, where the yellow group is the one inferred at the root.
