# Supplementary material for: The metabolic network of the last bacterial common ancestor
Source: Commun Biol. 2021 Mar 26;4:413. doi: 10.1038/s42003-021-01918-4 (PMC7997952; doi:10.1038/s42003-021-01918-4)
Supplement: Supplementary file 2 — Description of Additional Supplementary Files [file 42003_2021_1918_MOESM2_ESM.pdf]

Description of Additional Supplementary Files

File name: Supplementary Data 1

Description: List of genomes used in the analyses. A. GCF identifier according to NCBI, strain, taxonomic group, aerobic or anaerobic classification and genome size classification for all 5443 bacterial genomes downloaded from RefSeq. B. List of 1089 GCF identifiers for genomes used further in the analyses.

File name: Supplementary Data 2

Description: Presence of LBCA protein families in all taxonomic groups. For each group, the percentage of genomes where the family is present is shown.

File name: Supplementary Data 3

Description: Curated annotations of 146 LBCA protein families.

File name: Supplementary Data 4

Description: LBCA's metabolic network with target metabolites and non-universal reactions highlighted.

A. Reactions

B. Metabolites

File name: Supplementary Data 5

Description: Phylogenetic trees used in this study.

File name: Supplementary Data 6

Description: Sister Diversity Scores for 131 phylogenetic trees of LBCA protein families.

File name: Supplementary Data 7

Description: Verticality scores for LBCA protein families and corresponding metabolic reactions.
