# Supplementary material for: The metabolic network of the last bacterial common ancestor
Source: Commun Biol. 2021 Mar 26;4:413. doi: 10.1038/s42003-021-01918-4 (PMC7997952; doi:10.1038/s42003-021-01918-4)
Supplement: Supplementary file 10 — Reporting Summary [file 42003_2021_1918_MOESM10_ESM.pdf]

nature research

Corresponding author(s): Joana C. Xavier

Last updated by author(s): Feb 20, 2021

Reporting Summary

Nature Research wishes to improve the reproducibility of the work that we publish. This form provides structure for consistency and transparency in reporting. For further information on Nature Research policies, see our Editorial Policies and the Editorial Policy Checklist.

Statistics

For all statistical analyses, confirm that the following items are present in the figure legend, table legend, main text, or Methods section.

n/a	Confirmed	
☐	☒	The exact sample size (n) for each experimental group/condition, given as a discrete number and unit of measurement
☐	☒	A statement on whether measurements were taken from distinct samples or whether the same sample was measured repeatedly
☐	☒	The statistical test(s) used AND whether they are one- or two-sided<br>*Only common tests should be described solely by name; describe more complex techniques in the Methods section.*
☒	☐	A description of all covariates tested
☐	☒	A description of any assumptions or corrections, such as tests of normality and adjustment for multiple comparisons
☒	☐	A full description of the statistical parameters including central tendency (e.g. means) or other basic estimates (e.g. regression coefficient) AND variation (e.g. standard deviation) or associated estimates of uncertainty (e.g. confidence intervals)
☐	☒	For null hypothesis testing, the test statistic (e.g. F, t, r) with confidence intervals, effect sizes, degrees of freedom and P value noted<br>*Give P values as exact values whenever suitable.*
☒	☐	For Bayesian analysis, information on the choice of priors and Markov chain Monte Carlo settings
☒	☐	For hierarchical and complex designs, identification of the appropriate level for tests and full reporting of outcomes
☒	☐	Estimates of effect sizes (e.g. Cohen's d, Pearson's r), indicating how they were calculated

Our web collection on statistics for biologists contains articles on many of the points above.

Software and code

Policy information about availability of computer code

Data collection: All data sources and packages and their usage are described in the Methods with the corresponding versions and references (including NCBI, KEGG and JGI GOLD). New code used here consisted of batch subroutines to run multiple collections at once. No new software was developed for this purpose.

Data analysis: All packages used for data analysis are described in the methods with the corresponding versions and references (including BLAST, EMBOSS, MAFFT, RAxML, MCL, MAD, ETE3, PastML and Cytoscape). New code used here consisted of batch subroutines to run multiple analyses at once, calculations and statistical analysis as thoroughly described in the Methods. No new software was developed for this purpose.

For manuscripts utilizing custom algorithms or software that are central to the research but not yet described in published literature, software must be made available to editors and reviewers. We strongly encourage code deposition in a community repository (e.g. GitHub). See the Nature Research guidelines for submitting code & software for further information.

Data

Policy information about availability of data

All manuscripts must include a data availability statement. This statement should provide the following information, where applicable:

- Accession codes, unique identifiers, or web links for publicly available datasets
- A list of figures that have associated raw data
- A description of any restrictions on data availability

Sequence data that supports the findings of this study is available in NCBI RefSeq (GCF identifiers are provided in Supplementary Table 1). Metabolic data is available in KEGG. Phylogenetic trees and other relevant data are provided in Supplementary Datasets.

Field-specific reporting

Please select the one below that is the best fit for your research. If you are not sure, read the appropriate sections before making your selection.

☒ Life sciences ☐ Behavioural & social sciences ☐ Ecological, evolutionary & environmental sciences

For a reference copy of the document with all sections, see nature.com/documents/nr-reporting-summary-flat.pdf

Life sciences study design

All studies must disclose on these points even when the disclosure is negative.

Sample size	All bacterial genomes from NCBI, RefSeq version September 2016 were collected for the initial analysis, and those 1089 classified as anaerobic were retained for further analysis.
Data exclusions	No data was excluded from the analysis.
Replication	All attempts at replication were successful.
Randomization	No experimental groups were used in this study and therefore randomization does not apply.
Blinding	No experimental groups of subjects were used in this study and therefore blinding does not apply.

Reporting for specific materials, systems and methods

We require information from authors about some types of materials, experimental systems and methods used in many studies. Here, indicate whether each material, system or method listed is relevant to your study. If you are not sure if a list item applies to your research, read the appropriate section before selecting a response.

Materials & experimental systems

n/a	Involved in the study	
☒	☐	Antibodies
☒	☐	Eukaryotic cell lines
☒	☐	Palaeontology and archaeology
☒	☐	Animals and other organisms
☒	☐	Human research participants
☒	☐	Clinical data
☒	☐	Dual use research of concern

Methods

n/a	Involved in the study	
☒	☐	ChIP-seq
☒	☐	Flow cytometry
☒	☐	MRI-based neuroimaging
